# Changes of Gene Expression Patterns of Muscle Pathophysiology-Related Transcription Factors During Denervated Muscle Atrophy

**DOI:** 10.3389/fphys.2022.923190

**Published:** 2022-06-24

**Authors:** Xiaoming Yang, Ming Li, Yanan Ji, Yinghao Lin, Lai Xu, Xiaosong Gu, Hualin Sun, Wei Wang, Yuntian Shen, Hua Liu, Jianwei Zhu

**Affiliations:** ^1^ School of Biology and Basic Medical Sciences, Medical College of Soochow University, Suzhou, China; ^2^ Key Laboratory of Neuroregeneration of Jiangsu and Ministry of Education, NMPA Key Laboratory for Research and Evaluation of Tissue Engineering Technology Products, Co-Innovation Center of Neuroregeneration, Jiangsu Clinical Medicine Center of Tissue Engineering and Nerve Injury Repair, Nantong University, Nantong, China; ^3^ Department of Laboratory Medicine, Binhai County People’s Hospital affiliated to Kangda College of Nanjing Medical University, Yancheng, China; ^4^ Department of Orthopedics, Affiliated Hospital of Nantong University, Nantong, China; ^5^ Department of Orthopedics, Haian Hospital of Traditional Chinese Medicine, Nantong, China

**Keywords:** denervation, muscle atrophy, transcription factor, inflammation, transcriptome

## Abstract

Peripheral nerve injury is common, and can lead to skeletal muscle atrophy and dysfunction. However, the underlying molecular mechanisms are not fully understood. The transcription factors have been proved to play a key role in denervated muscle atrophy. In order to systematically analyze transcription factors and obtain more comprehensive information of the molecular regulatory mechanisms in denervated muscle atrophy, a new transcriptome survey focused on transcription factors are warranted. In the current study, we used microarray to identify and analyze differentially expressed genes encoding transcription factors in denervated muscle atrophy in a rat model of sciatic nerve dissection. Gene Ontology and Kyoto Encyclopedia of Genes and Genomes analyses were used to explore the biological functions of differentially expressed transcription factors and their target genes related to skeletal muscle pathophysiology. We found that the differentially expressed transcription factors were mainly involved in the immune response. Based on correlation analysis and the expression trends of transcription factors, 18 differentially expressed transcription factors were identified. Stat3, Myod1, Runx1, Atf3, Junb, Runx2, Myf6, Stat5a, Tead4, Klf5, Myog, Mef2a, and Hes6 were upregulated. Ppargc1a, Nr4a1, Lhx2, Ppara, and Rxrg were downregulated. Functional network mapping revealed that these transcription factors are mainly involved in inflammation, development, aging, proteolysis, differentiation, regeneration, autophagy, oxidative stress, atrophy, and ubiquitination. These findings may help understand the regulatory mechanisms of denervated muscle atrophy and provide potential targets for future therapeutic interventions for muscle atrophy following peripheral nerve injury.

## Introduction

Peripheral nerve injury inevitably causes neuronal degeneration, muscle atrophy, and fibrosis, to different extent. Peripheral nerve regeneration is slow (1 mm/d). Consequently, irreversible atrophy often occurs before skeletal muscle is reinnervated. This seriously affects the functional reconstruction of target muscle after nerve injury, with a resultant heavy burden on the patient and society (Gu et al., 2011; Sun et al., 2021).

Denervation of target muscle caused by lower motor neuron damage is accompanied by flaccid paralysis and rapid atrophy; reduction of muscle mass, strength, and muscle fiber diameter; and muscle fiber apoptosis (Siu and Alway, 2005). Skeletal muscle atrophy is not a degenerative process but reflects a change in the balance between protein synthesis and proteolysis in muscle fiber (Bruusgaard and Gundersen, 2008). The underlying mechanisms of denervated muscle atrophy are not fully understood, which hinders therapeutic progress in the field. Novel biological therapeutic targets for denervated muscle atrophy are urgently needed.

Two major protein degradation pathways, the ubiquitin–proteasome pathway and autophagy–lysosome pathway, are activated during muscle atrophy and lead to varying degrees of muscle mass loss (Schiaffino et al., 2013). Both pathways involve multiple atrophy-related genes regulated by specific transcription factors (TFs), whose activation is also controlled by specific signals. For example, the forkhead box O (FoxO) TFs are mainly negatively regulated by Akt (Stitt et al., 2004), while nuclear factor κB is mainly activated by inflammatory cytokines (Peterson et al., 2011). In recent years, many TFs have been identified that play an important role in muscle atrophy. Specifically, FoxO-like TFs are key mediators of the catabolic response during skeletal muscle atrophy, and their activation promotes the expression of Muscle atrophy F-box (MAFbx) and muscle RING-finger protein-1 (MuRF-1), two muscle-specific ubiquitin ligases, which leads to a dramatic loss of muscle mass (Romanello and Sandri, 2010). FoxO3 controls the transcription of autophagy-related genes, including *LC3* and *Bnip3*, and BNIP3 mediates the effect of FoxO3 on autophagy (Mammucari et al., 2007). Further, denervation activates Stat3–IL6 signaling in fibrolipogenic progenitor cells, thereby facilitating muscle fiber atrophy and fibrosis (Madaro et al., 2018). When skeletal muscle is disused, e.g., during immobilization and denervation, mitochondria undergo a series of deleterious changes, which eventually result in mitophagy and apoptosis cascade (Ji and Yeo, 2019). Overall, muscle atrophy disturbs the expression of many transcription factors.

Previously, we performed transcriptome sequencing analysis and proposed the transcriptional regulation mode of denervated rat tibialis anterior muscle atrophy. The cDNA microarray analysis showed four stages, including oxidative stress stage, inflammation stage, atrophy stage and atrophic fibrosis stage occurred within the period of 0.25 h–28 days post nerve injury (Shen et al., 2019). Subsequently, we performed transcriptional analysis of rat tibialis anterior muscle at 12 h-7 days after denervation to analyze differentially alternatively spliced genes, which provided a global view of alternative splicing in denervated skeletal muscle atrophy (Qiu et al., 2021). Meanwhile, a RNA-sequencing analysis of lncRNAs was performed using denervated muscle atrophy model. In this study, a co-expression network analysis of lncRNAs and mRNAs provided further insights into the biological processes associated with muscle atrophy (Hitachi et al., 2021). In order to systematically analyze transcription factors and obtain more comprehensive information of the molecular regulatory mechanisms in denervated muscle atrophy, a new transcriptome survey focused on transcription factors are warranted. Here, we screened the expression of genes encoding TFs during denervated muscle atrophy in a rat model using transcriptome analysis and bioinformatics. We then explored the biological functions of the known muscle pathophysiology-related TFs using Gene Ontology (GO) and Kyoto Encyclopedia of Gene and Genomes (KEGG) analyses. We thus identified the TFs that may play an important regulatory role in denervated muscle atrophy, with possible implications for future targeted therapy and prognosis of muscle atrophy.

## Materials and Methods

### Animal Experiment

The study was approved by the animal care guidelines of Nantong University and ethically approved by Jiangsu Administration Committee of Experimental Animals. Sprague–Dawley rats (*n* = 60, weighing ∼200 g) were provided by the Experimental Animal Center of Nantong University, China. Rat model of sciatic nerve dissection was used (Shen et al., 2019). The rats were randomly divided into the sham and model group (*n* = 3 per group). In the model group, rats were anesthetized with intraperitoneal injection of mixed narcotics (100 mg/kg ketamine plus 10 mg/kg xylazine). Then the sciatic nerve was exposed through an incision at the mid-thigh of the left hind limb for transection to leave a 10-mm long defect. In the sham group, the rats were subjected to similar surgical procedures without sciatic nerve transection. The tibialis anterior (TA) muscle was collected from rats in both groups at different time points (0.25 h, 0.5 h, 3 h, 6 h, 12 h, 24 h, 3 days, 14 d, 21 days, and 28 days) after surgery (Shen et al., 2019).

### Microarray Hybridization and Analysis

The tibialis anterior muscle sample at the different time points was homogenized, and RNA was extracted with RNasey Mini Kit (Qiagen, San Francisco, CA, United States) according to the manufacturer’s instructions. Microarray analysis was performed using an Agilent SurePrint G3 Rat GE (8 × 60K, Design ID: 028279), as previously described (Shen et al., 2019). Microarray analysis was performed on an Agilent Gene Chip platform and scanned by Agilent Scanner G2505C (Agilent Technologies). Data were extracted from scanned images using Agilent Feature Extraction Software (version 10.7.1.1, Agilent Technologies). The raw data were normalized by Genespring Software (version 13.1, Agilent Technologies). This dataset is available at the NCBI Gene Expression Omnibus (GEO) repository with GEO accession: GSE201025.

### Bioinformatics Analysis

The mRNA levels in the tibialis anterior muscle of rats in the model group were compared with that in the sham groups at each time point after surgery. Differentially Expressed Genes (DEGs) were defined by fold change (>1.5 or <−1.5) and FDR < 0.05. The upstream regulator analysis of all DEGs was performed using Ingenuity Pathway Analysis (IPA) and the upstream TFs at each time point were identified (*p* < 0.05). Then, DEGs encoding TFs were obtained with a fold change of overlap >1.5 and FDR < 0.05. Screening the known muscle-related genes by testing their association with the key terms ‘‘muscle’’ in the PubMed database (Supplementary Table S1), and DEGs encoding TFs related to the pathophysiology of skeletal muscle were identified. For the heatmap, gene expression has been z-score normalized, all DEGs/DEGs encoding TFs were classified according to the expression pattern.

### Functional Analysis

The Kyoto Encyclopedia of Genes and Genomes (KEGG) database and Gene Ontology (GO) category database were used for functional annotation of differentially expressed genes. Enrichment analysis of GO categories was performed by R clusterProfiler (v3.14.3) package, and enrichment analysis of pathways was tested upon hypergeometric distribution by R “phyper” function. Those GO categories with a false discovery rate (FDR) < 0.05 were considered as significant enriched. While pathways with a *p* < 0.05 were regarded as enriched. Only those GO categories or pathways contains ≥5 DEGs were kept for further analysis.

### Biological Network Analysis of Differentially Expressed Transcription Factors and Their Target Genes

For visual analysis of biological function network of differentially expressed TFs and their target genes related to pathophysiology of skeletal muscle, the edges were represented using Cytoscape 3.6 (The Cytoscape Consortium; http://cytoscape.org/, United States).

### Statistical Analysis

The relative change represents the difference between various model group and the sham group. Student’s t*-*test is used to test the significance (*p* value) of the difference between the two groups. Correction of the *p* values was performed in R according to Benjamini-Hochberg method. Then the different genes were screened according to fold change (>1.5 or <−1.5) and FDR < 0.05. Gene expression correlation between samples was calculated as the Pearson correlation coefficient. GO and KEGG analysis were performed to calculate enrichment *p*-values using hypergeometric distribution tests.

## Results

### Changes in the Expression of Transcription Factor Genes During Denervated Muscle Atrophy

**FIGURE 1:**
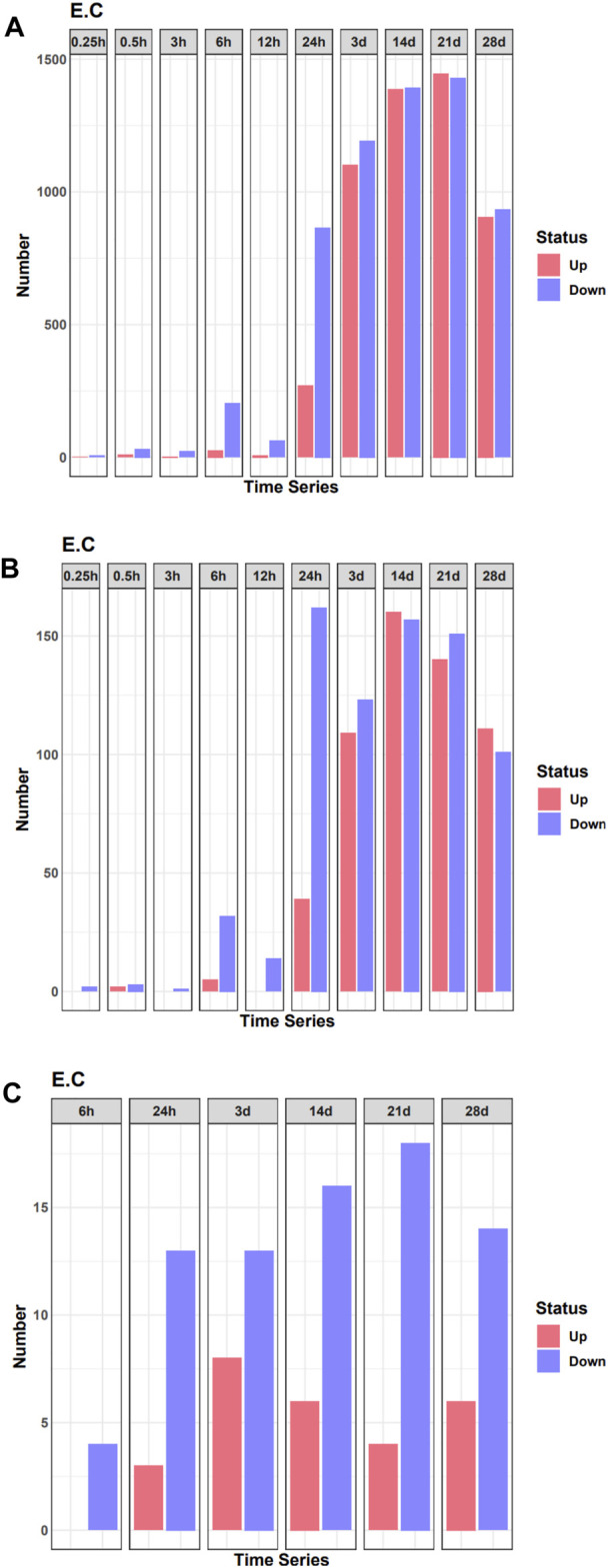
Quantification of differentially expressed genes (DEGs) in the denervated and sham groups at each time point examined. **(A)** The number of DEGs at each time point. **(B)** The number of DEGs of transcription factors (TFs) at each time point. **(C)** The number of differentially expressed TF genes related to the pathophysiology of skeletal muscle at each time point.

To analyze changes in the expression of TF during denervated muscle atrophy, we quantified DEGs in skeletal muscle in model and sham groups at different time points, as indicated. The number of DEGs began to increase significantly 24 h after denervation (Figure 1A). Correspondingly, the number of DEGs encoding TFs also began to increase sharply 24 h after denervation (Figure 1B). We then quantified the number of DEGs encoding TFs related to the pathophysiology of skeletal muscle at each time point; the number began to increase significantly 24 h after denervation (Figure 1C). These findings indicate that the 24 h time point after denervation may be a critical turning point in skeletal muscle atrophy.

### Functional Analysis of Differentially Expressed Genes and Targets of Differentially Expressed Genes Encoding Transcription Factors

**FIGURE 2:**
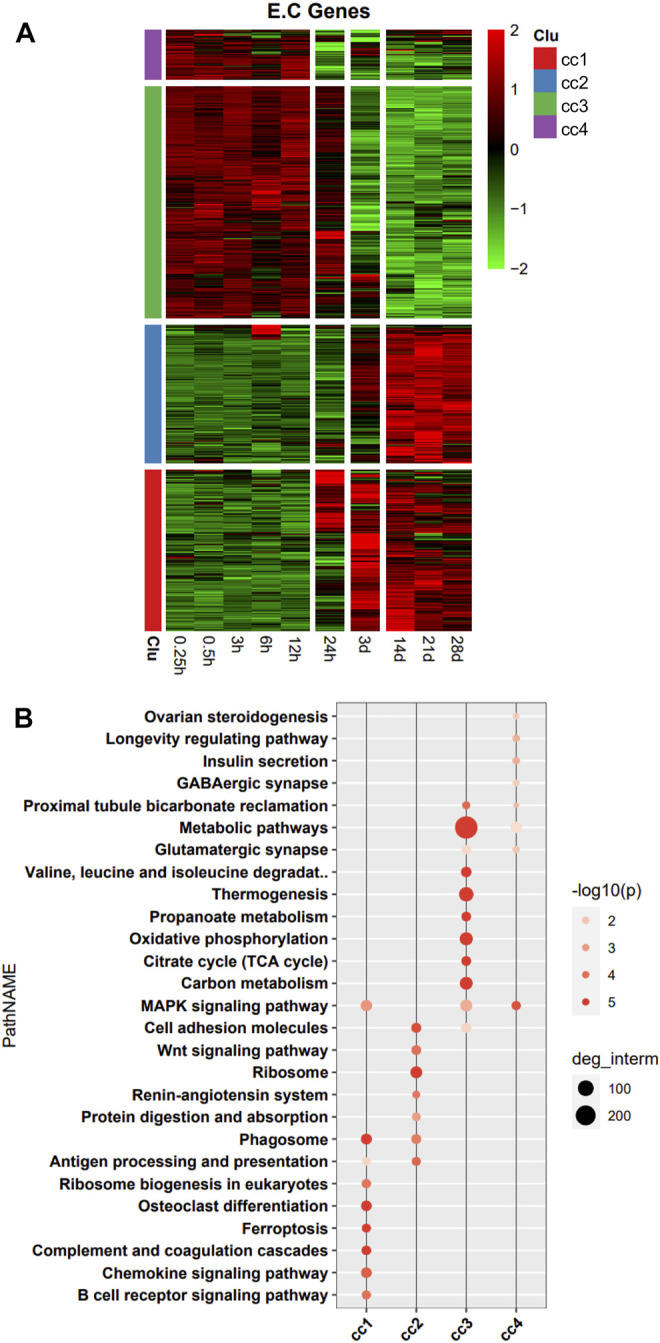
Heatmap and functional analysis of all differentially expressed genes (DEGs). **(A)** DEG expression patterns. **(B)** Functional enrichment map.

In the heatmap, DEG expression patterns fell into four clusters (cc1, cc2, cc3, and cc4). Cc1 and cc2 DEGs were upregulation with time; cc3 and cc4 DEGs were downregulation with time (Figure 2A). We also performed a functional analysis of DEGs in each cluster. We found that DEGs in cc1 were mainly involved in B cell receptor signaling pathway, chemokine signaling pathway, complement and coagulation cascade, ferroptosis, and phagosome. DEGs in cc2 were mainly involved in ribosome, cell adhesion, Wnt signaling pathway, phagosome, and antigen processing and presentation. DEGs in cc3 were mainly involved in metabolic pathways, oxidative phosphorylation, citrate cycle [tricarboxylic acid (TCA) cycle], carbon metabolism, and thermogenesis. DEGs in cc4 were mainly involved in MAPK signaling pathway and metabolic pathways (Figure 2B). Overall, DEGs in cc1 and cc2 were mainly involved in the immune response, while those in cc3 and cc4 were mainly involved in energy metabolism and oxidative phosphorylation.

**FIGURE 3:**
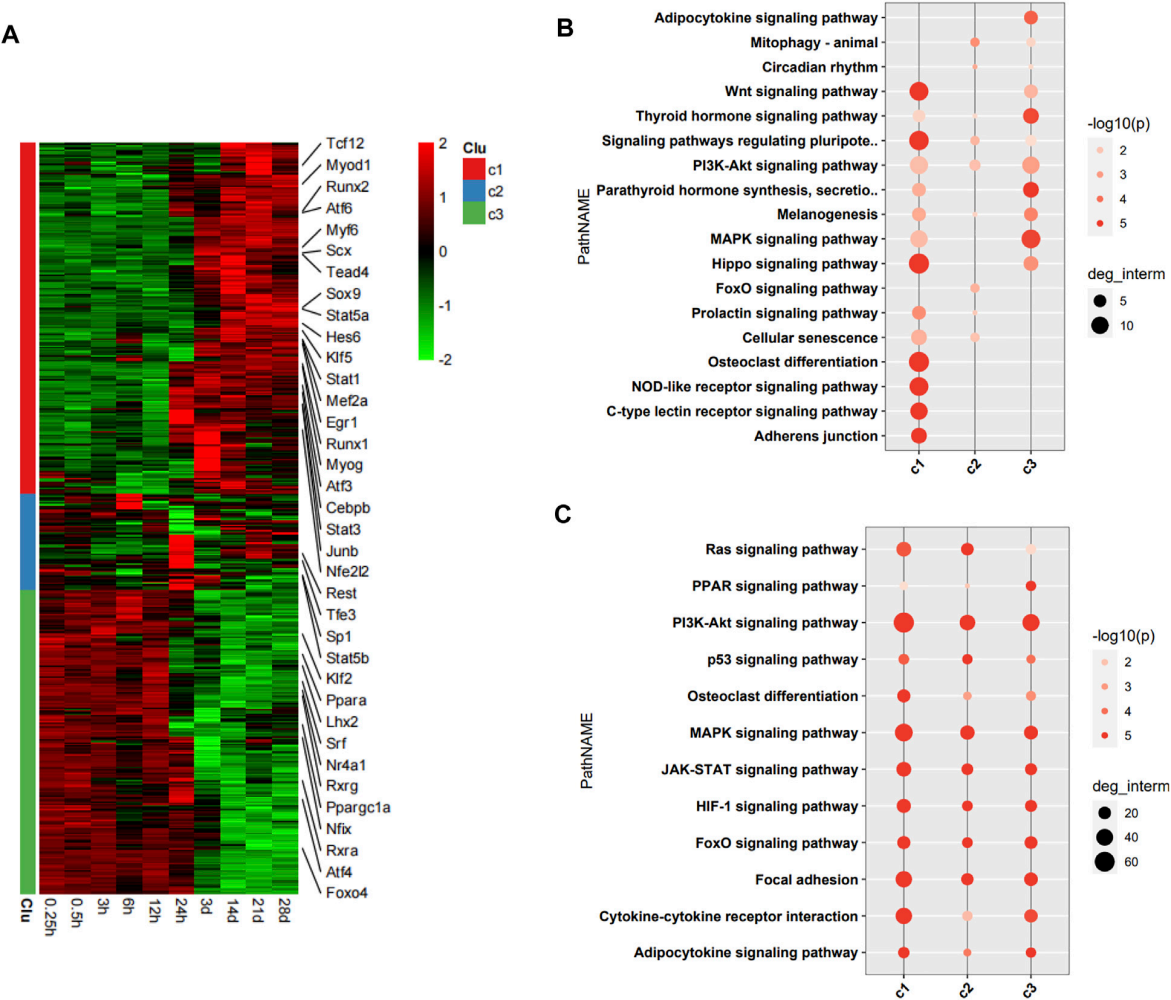
Functional analysis of differentially expressed transcription factors (TFs) and their target genes. **(A)** Heatmap of DEGs encoding TFs, with 36 TFs related to pathophysiology of skeletal muscle indicated on the right. **(B)** Enrichment analysis of target genes of differentially expressed TFs. **(C)** Enrichment analysis of target genes of differentially expressed TFs associated with the pathophysiology of skeletal muscle.

The DEGs encoding TFs were assigned to three clusters (c1, c2, and c3) in the heatmap (Figure 3A). 36 TFs encoded by DEGs related to the pathophysiology of skeletal muscle were indicated in the heatmap. The DEGs encoding TFs in c1 were upregulation over time, and those in c3 were downregulation over time. We then performed functional analysis of the target genes of differentially expressed TFs in each cluster. We found that the target genes of differentially expressed TFs in c1 were mainly involved in adherens junction, C-type lectin receptor signaling pathway, NOD-like receptor signaling pathway, osteoclast differentiation, Hippo signaling pathway, and Wnt signaling pathway. Those of TFs in c2 were mainly involved in FoxO signaling pathway, cellular senescence, and PI3K–Akt signaling pathway. Those of TFs in c3 were mainly involved in MAPK signaling pathway, thyroid hormone signaling pathway, and adipocytokine signaling pathway (Figure 3B). Functional analysis of the target genes of differentially expressed TFs in each cluster related to the pathophysiology of skeletal muscle revealed that the target genes in c1 were mainly involved in PI3K–Akt signaling pathway, MAPK signaling pathway, cytokine-cytokine receptor interaction, focal adhesion, JAK–STAT signaling pathway, HIF-1 signaling pathway, and FoxO signaling pathway. Those in c2 were mainly involved in PI3K–Akt signaling pathway, MAPK signaling pathway, focal adhesion, Ras signaling pathway, and JAK–STAT signaling pathway. Those in c3 were mainly involved in PI3K–Akt signaling pathway, MAPK signaling pathway, JAK–STAT signaling pathway, HIF-1 signaling pathway, FoxO signaling pathway, focal adhesion, and cytokine–cytokine receptor interaction (Figure 3C).

**FIGURE 4:**
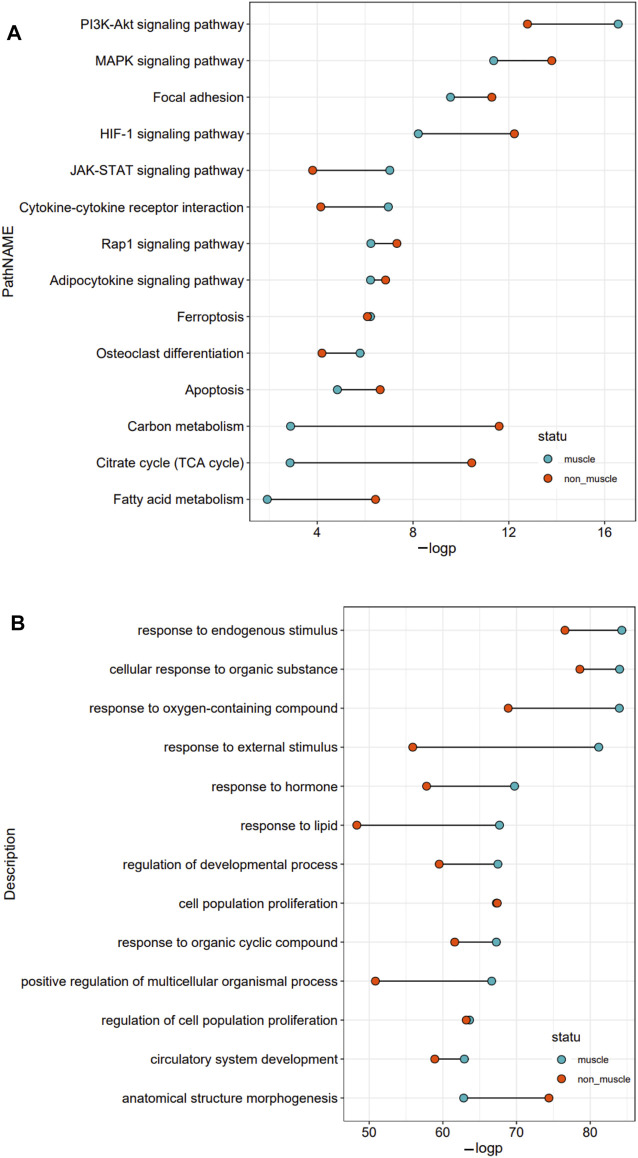
Functional comparison of target genes of differentially expressed TFs related and unrelated to the pathophysiology of skeletal muscle. **(A)** The union set of top 10 pathways in the two groups. **(B)** The union set of top 10 biological processes in the two groups.

To better understand the exact functions of target genes of muscle-related differentially expressed TFs, we performed a two-level functional comparison of target genes of muscle-related and non-muscle -related differentially expressed TFs. First, the union set of top 10 pathways which enriched in both groups was analyzed. The functional differences between the two groups of target genes mainly concerned carbon metabolism, fatty acid metabolism, TCA cycle, PI3K–Akt signaling pathway, HIF-1 signaling pathway, JAK–STAT signaling pathway, and cytokine–cytokine receptor interaction. Target genes of non-muscle atrophy-related differentially expressed TFs were more likely to be related to carbon metabolism, fatty acid metabolism, TCA cycle, and HIF-1 signaling pathway than the other gene set, while the target genes of muscle atrophy-related differentially expressed TFs were more likely to be related to PI3K–Akt signaling pathway, JAK–STAT signaling pathway, and cytokine–cytokine receptor interaction than the other gene set (Figure 4A). Then, the union set of 10 biological process terms which enriched in both groups was analyzed. The functional differences between the two groups of target genes concerned response to external stimulus, lipid, oxygen-containing compound, endogenous stimulus, and hormone, and positive regulation of multicellular organismal process. Further, the target genes of muscle atrophy-related differentially expressed TFs were more likely to be involved in these biological processes (Figure 4B) than the other genes. Overall, the target genes of non-muscle-related differentially expressed TFs are mainly involved in metabolism, while those of muscle-related differentially expressed TFs are mainly involved in the immune response.

### Functional Enrichment Analysis of Common Target of Differentially Expressed Genes Encoding Transcription Factors Related and Unrelated to Pathophysiology of Skeletal Muscle

**FIGURE 5:**
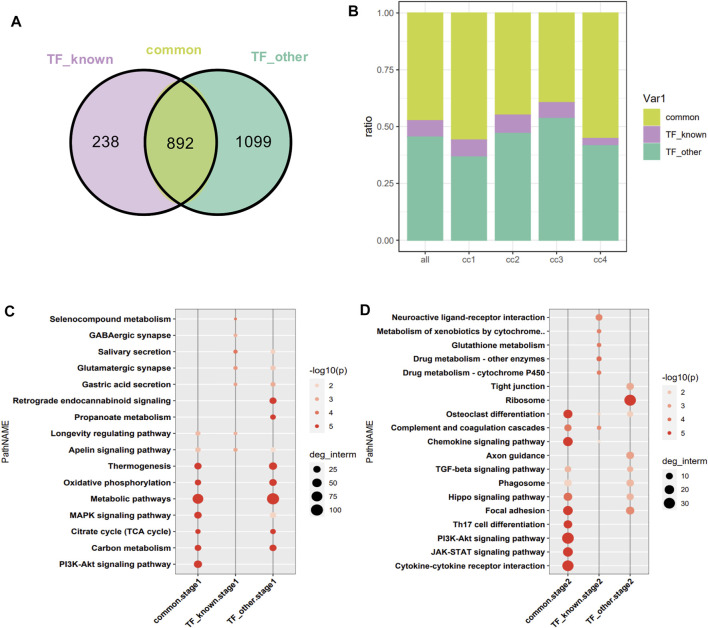
Intersection of targets of DEGs encoding TFs related and unrelated to the pathophysiology of skeletal muscle. **(A)** Venn diagram analysis. TF_known: specific target genes of DEGs encoding TFs related to the pathophysiology of skeletal muscle; TF_other: specific target genes of differentially expressed TFs unrelated to the pathophysiology of skeletal muscle. **(B)** Distribution of target genes in the four clusters. The horizontal axis represents the cluster to which the target gene belongs, with all as the reference. **(C)** Functional enrichment analysis of target genes at stage1. **(D)** Functional enrichment analysis of target genes at stage2.

1,130 target genes of differentially expressed TFs related and 1991 unrelated to the pathophysiology of skeletal muscle were analyzed. As shown by the constructed Venn diagram, we identified 892 target genes shared by the two groups (common), 238 specific target genes of differentially expressed TFs related to the pathophysiology of skeletal muscle (TF_known), 1,099 specific target genes of other differentially expressed TFs (TF_other) (Figure 5A). However, three group of target genes had no bias in the four clusters of DEGs (Figure 5B). Then, functional enrichment analysis of target genes in the cluster was performed in two stages (cc1, cc2-stage2, cc3, cc4-stage1). In stage1, the shared target genes were mainly associated with metabolic pathways, MAPK signaling pathway, Pl3K–Akt signaling pathway, carbon metabolism, thermogenesis, oxidative phosphorylation, and TCA cycle. The specific target genes in TF_known group were mainly related to salivary secretion, glutamatergic synapse, gastric acid secretion, longevity regulating pathway, and apelin signaling pathway. The specific target genes in TF_other group were mainly involved in metabolic pathways, oxidative phosphorylation, TCA cycle, thermogenesis, retrograde endocannabinoid signaling, and carbon metabolism (Figure 5C). In stage2, the shared target genes were mainly related to cytokine–cytokine receptor interaction, JAK–STAT signaling pathway, PI3K–Akt signaling pathway, Th17 cell differentiation, focal adhesion, osteoclast differentiation, chemokine signaling pathway, and Hippo signaling pathway. The target genes in TF_known group were mainly involved in neuroactive ligand–receptor interaction, metabolism of xenobiotics by cytochrome, glutathione metabolism, drug metabolism–cytochrome P450, and complement and coagulation cascades. The target genes in TF_other were mainly associated with ribosome, focal adhesion, axon guidance, phagosome, Hippo signaling pathway, and TGF-β signaling pathway (Figure 5D). The enrich pathways of shared targets is similar with that of all DEGs, which indicated that the shared target genes are critical in muscle atrophy.

**FIGURE 6:**
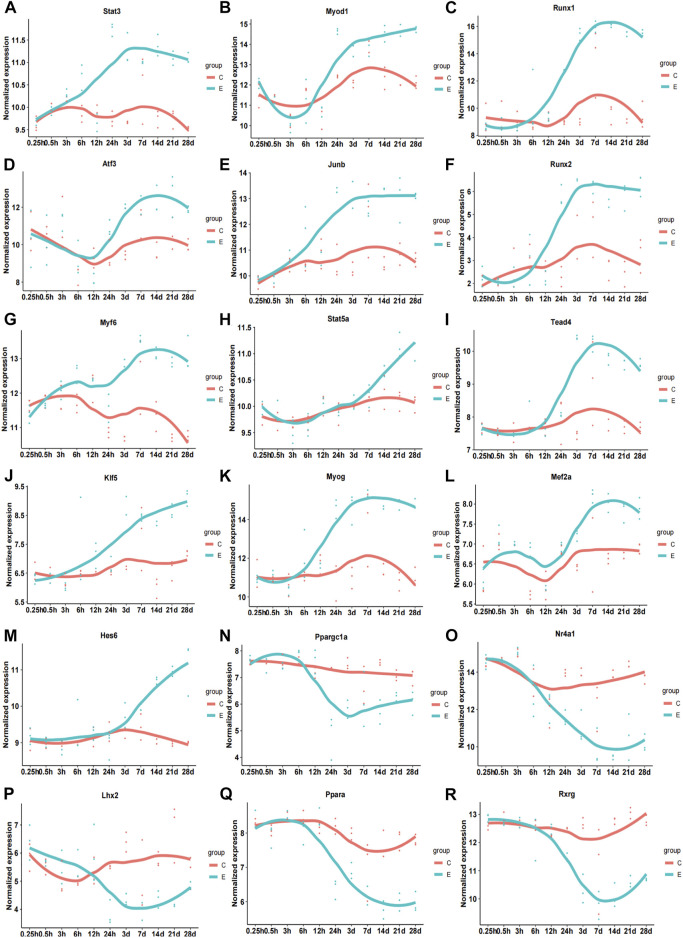
Time-dependent expression of differentially expressed genes of 18 transcription factors related to pathophysiology of skeletal muscle. (A) Stat3. (B) Myod1. (C) Runx1. (D) Atf3. (E) Junb. (F) Runx2. (G) Myf6. (H) Stat5b. (I) Tead4. (J) Klf5. (K) Myog. (L) Mef2a. (M) Hes6. (N) Ppargc1a. (O) Nr4a1. (P) Lhx2. (Q) Ppara. (R) Rxrg. C, sham group; E, denervation group.

Subsequently, we analyzed the correlation between differentially expressed TFs and their target genes in common-stage1 and common-stage2. Overall, 32 and 35 TFs were identified at stage 1 and stage 2, respectively (Table 1). Then, 32 TFs were identified at both stages, and we plotted the expression levels of genes for these shared 32 TFs over time (Supplementary Figures S1, S2). Among them, the expression of 18 TFs significantly upregulated/downregulated overtime, which suggested the TFs were closely related to skeletal muscle atrophy (Figure 6). *Stat3, Myod1, Runx1, Atf3, Junb, Runx2, Myf6, Stat5a, Tead4, Klf5, Myog, Mef2a,* and *Hes6* were upregulated; and *Ppargc1a, Nr4a1, Lhx2, Ppara,* and *Rxrg* were downregulated.

**TABLE 1:** Correlation between differentially expressed TFs and their target genes in common-stage1 and common-stage2.

Common-stage 1			Common-stage 2		
Gene	Negative	Positive	Gene	Negative	Positive
Stat3	32	0	Stat3	0	52
Srf	0	24	Cebpb	0	45
Stat5a	23	0	Myod1	0	33
Klf2	0	22	Srf	32	1
Runx1	22	0	Ppargc1a	30	0
Ppargc1a	0	21	Nfe2l2	0	31
Cebpb	18	0	Runx1	0	31
Myod1	18	0	Atf3	0	23
Foxo4	0	16	Atf4	22	0
Nfe2l2	15	0	Junb	0	22
Atf4	0	12	Runx2	0	20
Egr1	11	0	Myf6	0	19
Junb	11	0	Klf2	19	0
Myf6	11	0	Egr1	0	17
Runx2	10	0	Sox9	0	17
Tead4	10	0	Stat5a	0	17
Atf6	8	0	Tead4	0	17
Klf5	8	0	Foxo4	8	0
Mef2a	6	0	Nr4a1	8	0
Atf3	5	0	Klf5	0	6
Nfix	1	2	Atf6	0	5
Nr4a1	0	3	Myog	0	5
Sox9	3	0	Nfix	3	1
Tcf12	3	0	Mef2a	0	3
Ppara	0	2	Sp1	0	2
Hes6	1	0	Tcf12	0	2
Lhx2	0	1	Tfe3	0	2
Myog	1	0	Hes6	0	1
Rest	1	0	Rest	0	1
Rxrg	0	1	Lhx2	1	0
Sp1	0	1	Ppara	1	0
Tfe3	1	0	Rxrg	1	0
			Scx	0	4
			Stat1	0	1
			Stat5b	0	1

Stage1- cc3, cc4 (DEGs were downregulation with time); stage2- cc1, cc2 (DEGs were upregulation with time).

### Biological Function Network of Differentially Expressed Genes Encoding Transcription Factors and Their Target Genes Related to Pathophysiology of Skeletal Muscle

**FIGURE 7:**
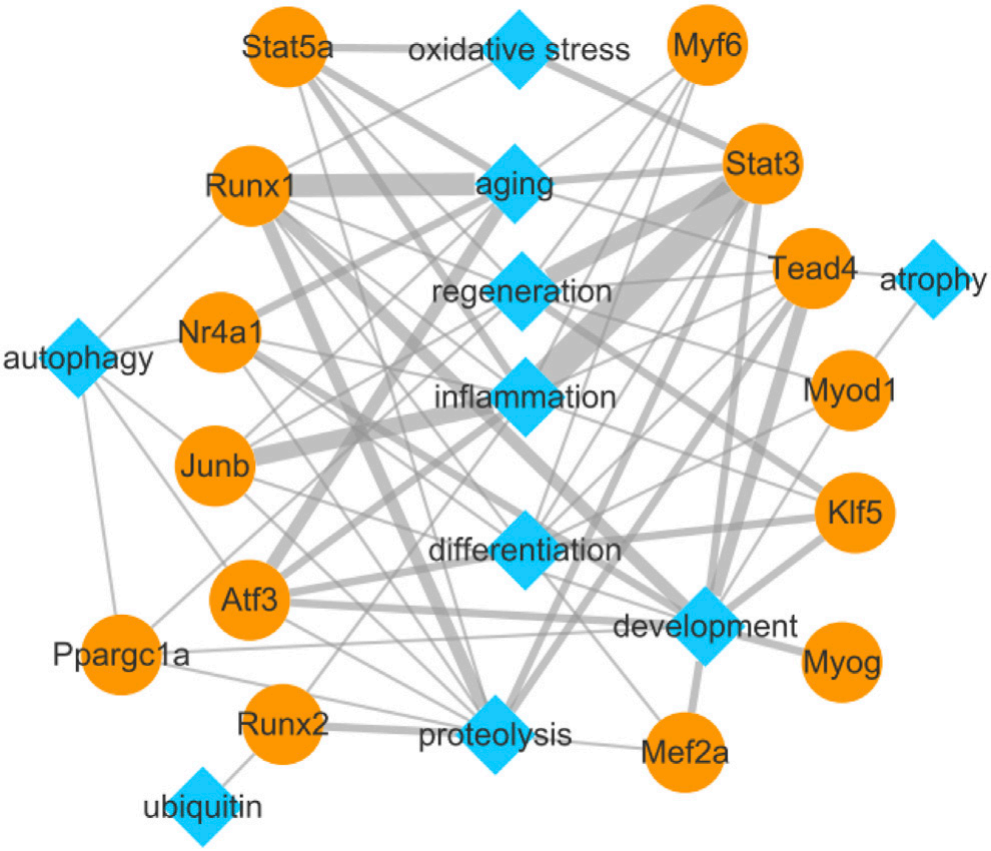
Biological function network of differentially expressed transcription factors and their target genes related to pathophysiology of skeletal muscle.

To explore the relationship between the identified TFs and biological functions in detail, we constructed a network diagram (Figure 7). The analysis revealed *Stat3, Junb, Stat5a, Atf3, Runx1, Nr4a1, Tead4, Myf6, Runx2,* and *Klf5* encoding inflammation-related TFs; *Tead4, Runx1, Stat3, Myog, Nr4a1, Klf5, Mef2a, Atf3, Ppargc1a, Myod1,* and *Junb* encoding development-related TFs; *Runx1, Atf3, Nr4a1, Stat5a, Stat3, Myf6,* and *Junb* encoding aging-related TFs; *Runx1, Stat3, Tead4, Runx2, Stat5a, Nr4a1, Junb, Atf3, Ppargc1a,* and *Mef2a* encoding proteolysis-related TFs; *Atf3, Klf5, Stat3, Myf6, Myod1, Junb, Tead4, Runx1, Mef2a,* and *Nr4a1* encoding differentiation-related TFs; *Stat3, Klf5, Stat5a, Runx1, Myf6, Myod1, Tead4, Ppargc1a,* and *Junb* encoding regeneration-related TFs; *Ppargc1a, Runx1, Atf3, Nr4a1,* and *Junb* encoding autophagy-related TFs; *Stat3, Stat5a,* and *Runx1* encoding oxidative stress-related TFs; *Tead4* and *Myod1* encoding atrophy-related TFs; and *Runx2* encoding ubiquitin-related TF.

## Discussion

Skeletal muscle atrophy is a typical manifestation of peripheral nerve injury, and involves complex pathogenic mechanisms. Recently, several studies have used whole transcriptomics to analyze differentially expressed mRNAs and lncRNAs in the process of muscle atrophy, providing a substantial amount of gene expression data for the study of muscle atrophy (Ehmsen et al., 2019; Hitachi et al., 2021; Qiu et al., 2021). In the current study, we identified TFs involved in skeletal muscle pathophysiology in a rat model of sciatic nerve dissection. The number of DEGs encoding TFs began to increase sharply 24 h after denervation. GO and KEGG analyses revealed the target genes of muscle-unrelated differentially expressed TFs are mainly involved in metabolism, while those of muscle-related differentially expressed TFs are mainly involved in the immune response. Then, functional enrichment analysis of common target of DEGs encoding TFs related and unrelated to muscle showed the shared target genes are critical in muscle atrophy. Correlation analysis between TFs and target genes in common, and we identified 18 significantly differentially expressed TFs related to the pathophysiology of skeletal muscle (upregulated: *Stat3, Myod1, Runx1, Atf3, Junb, Runx2, Myf6, Stat5a, Tead4, Klf5, Myog, Mef2a, Hes6*; downregulated: *Ppargc1a, Nr4a1, Lhx2, Ppara, Rxrg*). The biological functions of these TFs mainly include inflammation, development, aging, proteolysis, differentiation, regeneration, autophagy, oxidative stress, atrophy, and ubiquitination. These findings provide clues on the molecular regulatory mechanisms in skeletal muscle atrophy, with possible implications for the identification of new therapeutic targets.

### Transcription Factors Involved in Inflammation

The identified TFs involved in inflammation mainly included *Stat3, Stat5a, JunB,* and *Atf3*. *Stat3* is as a component of IL-6–activated acute phase response factor complex and plays a crucial role in stimulating expression of innate immune mediators in the liver (Hillmer et al., 2016). Our previous works have shown Aspirin could alleviate denervation-induced muscle atrophy, and inhibits type I–II muscle fiber transition and mitophagy by regulating the Stat3 inflammatory signaling pathway and the Sirt1/PGC1α axis (Wan et al., 2020); IL-6/JAK/STAT3 signaling pathway is strongly activated during denervated muscle atrophy (Sun et al., 2021); High IL-6 levels aggravated C2C12 myotube atrophy by activating JAK/Stat3, while ruxolitinib (JAK1/2 inhibitor) or C188-9 (Stat3 inhibitor) attenuated IL-6–induced C2C12 myotube atrophy (Huang et al., 2020). Stat5a and Stat3 are both key players in T-cell differentiation and homeostasis. *Stat3* is crucial for Th17 differentiation and suppresses Treg cells (Wei et al., 2008). Conversely, Stat5a is essential for Treg cell development and maintenance, and negatively regulates Th17 cell differentiation. Moreover, Stat5a is required for Th2 cell differentiation and allergic airway inflammation (Takatori et al., 2005). Collectively, Stat3 is upregulated during denervated muscle atrophy, and Stat5a and Stat3 are both closely related to the immune response, suggesting that Stat5a and Stat3 may play an important role in denervated muscle atrophy. JunB is an activator protein-1 (AP-1d) TF subunit, and a main determinant of muscle growth or atrophy in adults. Reduction of *JunB* expression in adult muscle by RNA interference leads to muscle atrophy, whereas *JunB* transfection of denervated muscle prevents fiber atrophy. Therefore, JunB is necessary for muscle size maintenance, rapid induction of hypertrophy and blocking atrophy (Raffaello et al., 2010). However, in the current study, JunB was up-regulated during denervated muscle atrophy, which was consistent with the findings of Salem et al. (2010). Overall, the role of JunB in denervated muscle atrophy remains to be further explored. Atf3 is a stress-induced TF that regulates immune and metabolic homeostasis (Zhu et al., 2018). Atf3 was detected in the skeletal muscle and spinal cord motor neurons in paralyzed mouse with amyotrophic lateral sclerosis (Gonzalez de Aguilar et al., 2008). Atf3 is a key regulator of local and systemic inflammation. It negatively regulates the expression of inflammatory genes in immune cells while positively regulating the expression of inflammatory genes in non-immune cells (Hai et al., 2010), suggesting that high Atf3 expression is associated with inflammation. The findings presented herein indicate that Stat3, Stat5a, JunB, and Atf3 genes are upregulated during denervated muscle atrophy, that is, these TFs may influence the development of muscle atrophy by involvement in inflammatory responses. Further exploration of the exact mechanism is warranted.

### Transcription Factors Involved in Development

The identified TFs involved in development mainly included *Runx1, Stat3, Myog, Nr4a1, Klf5, Mef2a* and *Tead4*. *Runx1* is a highly-conserved TF that regulates embryonic development, angiogenesis, hematopoiesis, immune response, and inflammatory response (Tang et al., 2018). It also is a determinant of the proliferation and differentiation of multiple cell types during development and adulthood (Koth et al., 2020). The expression level of *Runx1* is very low in adult muscle tissue but is detected in ischemic muscle, and therefore, plays a role in muscle response to injury (Bao et al., 2018). *Runx1* overexpression inhibits myogenic differentiation and promotes myoblast proliferation in C2C12 cells (Bao et al., 2018). Herein, *Runx1* is upregulated in denervated muscle atrophy, but its specific role in the process requires further investigation. STAT3 is a key regulator of multiple types of muscle-resident cells, including muscle stem cells (MuSCs), myofibers, and macrophages (Sala and Sacco, 2016). IL-6–activated Stat3 signaling modulates muscle satellite cell behavior and promotes myogenic lineage progression by regulating Myod1 (Tierney et al., 2014). The Jak2/Stat3 pathway is involved in lysine deficiency-induced apoptosis and inhibition of skeletal muscle growth (Song et al., 2020). STAT3 is a node that integrates multiple signaling pathways in the skeletal muscle (Sala and Sacco, 2016), and therefore, its role in skeletal muscle atrophy may be very complex. *Myog* is one of the master regulators of skeletal muscle lineage development and pluripotent stem cell differentiation, and plays an important role in muscle development (Esteves de Lima and Relaix, 2021). It is a muscle-specific TF and its expression is significantly elevated in denervated skeletal muscle. Our recent study suggested inhibiting Myog expression significantly alleviates denervation-induced skeletal muscle atrophy, which is accompanied by decreased expression of MuRF1 and MAFbx (Ma et al., 2021). These observations suggest that Myog plays a complex role in denervated skeletal muscle. *Nr4a1* is a myogenic factor involved in muscle development and regeneration. Further, muscle-specific deletion of *Nr4a1* impairs muscle growth (Pan et al., 2019; Liu Y. et al., 2021). *Nr4a1* overexpression accelerates myoblast differentiation and fusion of C2C12 cells. Conversely, inhibiting *Nr4a1* expression inhibits the differentiation and fusion of C2C12 cells (Pan et al., 2019). Magnusson et al. showed that *Nr4a1* expression is downregulated during denervated muscle atrophy, which is consistent with the findings of the current study (Magnusson et al., 2005). Downregulation of *Nr4a1* expression during denervated muscle atrophy may be related to its involvement in inhibiting the differentiation and fusion of muscle stem cells, but the specific molecular mechanism needs further study. *Klf5* is a zinc finger TF that is crucial for skeletal muscle development and regeneration (Zhang D.-H. et al., 2020). Differential gene and functional enrichment analyses revealed that *Klf5* affects the development of satellite cells in chicken skeletal muscle by regulating the expression of myogenic factor family and myosin family members, and other myogenic factors, and regulates atrophy of chicken skeletal muscle *via* the Wnt/β-catenin protein signaling pathway (Zhang D.-H. et al., 2020). KLF5 is upregulated in atrophic myotubes as part of an early response to dexamethasone or simulated microgravity *in vitro*. Selective *Klf5* deletion dramatically relieves skeletal muscle atrophy caused by de-mechanical loading in mouse. Further, Am80, a KLF5 inhibitor, inhibits dexamethasone- and microgravity-induced muscle atrophy *in vitro*, and oral Am80 improves disuse- and dexamethasone-induced muscle atrophy in mouse (Liu L. et al., 2021). Therefore, KLF5 is a key TF that mediates muscle atrophy and Am80 could be a potential preventive treatment drug. *Mef2a,* member of the Mef2 family, belongs to the MADS-box superfamily and is involved in a variety of cellular processes, including neuronal differentiation, muscle development, cell growth control, and apoptosis (Xiong et al., 2019). Mef2a activates many muscle-specific, growth factor-inducible, and stress-inducible genes (Wu et al., 2018). In denervated skeletal muscle, Mef2a regulates calpain 3 gene expression, which subsequently leads to the activation of Ca^2+^-regulated proteolytic enzymes, resulting in skeletal muscle atrophy (Wu et al., 2018). MEF2A also acts as a PINCH TF in neuroinflammation: TNFα-mediated activation of MEF2A *via* cellular Ca^2+^-induced PINCH activation, leading to the disruption of PINCH–ILK–parvin ternary complex and actin depolymerization (Natarajaseenivasan et al., 2020). *Tead4* is an important Tead family member that functions as a downstream effector of the Hippo pathway and is involved in cell proliferation and survival, tissue regeneration, and stem cell maintenance (Chen et al., 2020). Tead4 levels are high in the skeletal muscle tissue in mouse model of acute sepsis. Upregulation of miR-351 inhibits *Tead4* expression blocking the Hippo signaling pathway, thereby inhibiting degradation of skeletal muscle proteins and atrophy of the skeletal muscle in this mouse model (Zhang et al., 2018). In summary, upregulated TFs *Runx1, Stat3, Myog, Klf5, Tead4,* and *Mef2a,* and downregulated TF *Nr4a1* are all involved in muscle development. However, further experimental verification is needed to test how they participate in denervated muscle atrophy.

### Transcription Factors Involved in Aging

The identified TFs involved in aging mainly included *Runx1, Atf3, Nr4a1, Stat5a,* and *Stat3.* Sarcopenia, a progressive decline in muscle mass and strength that occurs with aging, is the most common type of muscle atrophy in humans. Imbalance of proteostasis, mitochondrial dysfunction and inflammatory disturbances are main intracellular mechanisms causes for sarcopenia, in addition to the change of muscle fiber composition, neuromuscular drive, hormones and muscle satellite cell (Wiedmer et al., 2021). The expression of Runx1 increased substantially from 15 to 24 month in quadriceps muscles of female mice, month age coincided with the transition to sarcopenia (Barns et al., 2014). Long-term resistance wheel exercise could reduce *Runx1* mRNA expression significantly in female mice compared with sedentary controls at 23 months, as well as improved some markers of the mitochondrial and autophagosomal pathways (White et al., 2016). As an activator protein-1 TF, *Atf3* is essential for remodeling chromatin accessibility to promote cellular senescence (Zhang et al., 2021). Genotoxic-responsive transcription factors ATF3 upregulated in motor neurons, which may contribute to neuronal aging and motor neuron vulnerability in human neuromuscular disorders (de Waard et al., 2010). ATF3 also drives cell senescence through TGFβ/Pdcd5 pathway in myocardium (Huang et al., 2022). The expression of ATF3 is upregulated during denervated muscle atrophy, which may be related to its effect on cell senescence in skeletal muscle. *Nr4a1* is a nutrient sensor that triggers mitochondrial biogenesis and improves mitochondrial intrinsic function. The *Nr4a* subfamily is a potential therapeutic target for aging-related diseases (Paillasse and de Medina, 2015). In skeletal muscle, *Nr4a1* (Nur77) is the most highly expressed Nr4a isoform and is selectively expressed in type 2 glycolytic myofibers, the type of atrophic myofibers preferential observed preferential in aging (Cortez-Toledo et al., 2017). Herein, the expression of *Nr4a1* is downregulated during denervated muscle atrophy, which may be related to its effect on mitochondrial function. The exact mechanism remains to be determined. Endogenous STAT5a represses gene expression at three mitochondrial DNA promoters by binding to the transcriptional control region of mitochondrial DNA (Chueh et al., 2020), and may be thereby involved in the aging process. Constitutively active *Stat5a* induces p53- and retinoblastoma-dependent cellular senescence responses (Mallette et al., 2007). Genetic and pharmacological inhibition of *Stat3* signaling improves stem cell homeostasis and physiology in aging and dystrophic muscle (Chazaud and Mouchiroud, 2014). Further studies are needed to investigate whether upregulation/downregulation of the TFs in denervated muscle atrophy is associated with senescence and an aging phenotype in skeletal muscle.

### Transcription Factors Involved in Proteolysis

The identified TFs involved in proteolysis mainly included *Stat3, Runx1,* and *Runx2.* Chronic activation of STAT3 in muscle fiber promotes protein degradation, which leads to skeletal muscle atrophy. Transient pharmacological inhibition of *Stat3* improves skeletal muscle atrophy in various pathologies (Sala and Sacco, 2016). Inhibition of *Stat3* activation represses caspase-3 expression and the ubiquitin–proteasome system, thereby preserving muscle mass in cancer cachexia (Silva et al., 2015). TTI-101 is a Stat3 inhibitor that blocks muscle proteolysis in rat model of chronic kidney disease (Zhang L. et al., 2020). Therefore, *Stat3* may be involved in skeletal muscle atrophy by activating the proteolytic pathway. RUNX1 is seldom expressed in innervated muscle but highly expressed in denervated muscle (Wang et al., 2005). One week after neuromuscular communication is impaired, increased *Runx1* expression, and decreased wet muscle mass and muscle fiber cross-sectional area (CSA) are observed (Hunt et al., 2022). It remains to be determined how increased *Runx1* expression promotes proteolysis and causes skeletal muscle atrophy. *Runx2* promotes TGF-β signaling, indicating its crucial role in fibrosis. Further, activation of TGF-β signaling activates proteolytic pathways, thereby leading to skeletal muscle atrophy (Kim et al., 2013; Abrigo et al., 2018). Therefore, *Runx2* regulates TGF-β signaling to activate proteolysis and participate in the process of muscle atrophy. Proteolysis is one of the typical features of muscle atrophy. *Stat3, Runx1,* and *Runx2* are closely related to the proteolytic process, and their roles in muscle atrophy should be further explored.

### Transcription Factors Involved in Differentiation

The identified TFs involved in differentiation mainly included *Atf3, Klf5, Stat3, Myf6,* and *Myod1*. *Atf3*, an upstream regulator of pro-inflammatory smooth muscle cell, is involved in the transition of diseased smooth muscle cell to initial atherosclerosis, and restoration of Atf3 activity prevents dedifferentiation of vascular smooth muscle cell (Wang et al., 2021). *Klf5* is an important regulator of skeletal muscle differentiation that regulates muscle differentiation by directly targeting muscle-specific genes in mouse (Hayashi et al., 2016). *Klf5* exerts multiple effects on cell differentiation and is a target of multiple miRNAs in smooth muscle (Joshi et al., 2012). STAT3 promotes myogenic lineage progression of muscle stem cell, and its inhibition promotes symmetrical expansion of muscle stem cell (Sala and Sacco, 2016). *Stat3* induces differentiation of muscle stem cell by interacting with myoD (Yang et al., 2009). Nonthermal atmospheric plasma treatment enhances myoblast differentiation by triggering Stat3 phosphorylation (Park et al., 2019). Myogenic differentiation factors (*Myog, Myod1*, and *Myf6*) are upregulated early in denervated muscle atrophy, suggesting their roles in initial atrophy (Gronholdt-Klein et al., 2019). *Myf6* is a member of the bHLH myogenic regulatory TF family (Hinits et al., 2007). *Myf6* establishes ligand/receptor interactions between muscle stem cell and the associated muscle fiber, and transcriptionally regulates a variety of myokines and secreted proteins in skeletal muscle fiber (Lazure et al., 2020). During C2C12 myoblast differentiation, miR-374b specifically binds to the 3′ untranslated region of *Myf6* and downregulates Myf6 expression at mRNA and protein levels (Ma et al., 2015). SETD3 is a histone methyltransferase that is abundantly expressed in muscle tissues. SETD3 overexpression activates the transcription of muscle-related genes, myogenin, muscle creatine kinase, and Myf6, thereby inducing muscle cell differentiation (Eom et al., 2011). *Myod1* is important for muscle cell differentiation (Huang et al., 2016). It is a key player in muscle-specific enhancer–promoter communication, coordinating the regulation of muscle fiber size (Rovito et al., 2021). Muscle enhancers are regulated *via* coordinated binding of TFs, including c-Jun, Jdp2, Meis, and Runx1. These TFs are recruited to muscle enhancers in a Myod1-dependent manner. *Myod1* and enhancer-related TFs cooperate to assemble and regulate enhancers, thereby enhancing the expression of muscle-related genes (Blum and Dynlacht, 2013). Further studies on the specific mechanisms underpinning the roles of *Atf3, Klf5, Stat3, Myf6,* and *Myod1* in denervated muscle atrophy are warranted.

### Transcription Factors Involved in Autophagy

The identified TFs involved in autophagy were *Ppargc1a, Runx1, Atf3, Nr4a1,* and *JunB. Ppargc1a,* encoding PGC1α, is a member of the *Ppargc1* transcriptional co-activator family that controls mitochondrial biogenesis by co-activating major TFs that regulate mitochondrial genes. It is a central organizer of metabolic function, oxidative state, and mitochondrial biogenesis and function (Salazar et al., 2020). Pyrroloquinoline quinone inhibits denervated skeletal muscle atrophy by activating PGC1α and integrating mitochondrial electron transport chain complexes (Kuo et al., 2015). PGC1α protects against skeletal muscle atrophy by inhibiting FoxO3 and suppressing transcription of atrophy-related genes (Sandri et al., 2006). Elevated PGC1α activity contributes to maintaining mitochondrial biogenesis and muscle function (Da Cruz et al., 2012). Further, PGC1α upregulates autophagy *via* a SQSTM1-dependent mechanism, thereby reducing senescence (Salazar et al., 2020). Ppargc1a overexpression *in vivo* inhibits mitophagy enhanced skeletal muscle with aging (Yeo et al., 2019). In the present study, *Ppargc1a* gene expression was downregulated in denervated muscle atrophy, suggesting that *Ppargc1a* involved in denervated muscle atrophy by regulating mitophagy. *Runx1* is essential for muscle homeostasis, by preventing myofibril disorder and autophagy in denervated muscle fiber (Wang et al., 2005). It therefore likely inhibits autophagy. Nonetheless, we observed significant activation of mitophagy during denervated muscle atrophy, suggesting that *Runx1* plays complex roles in denervated muscle atrophy, which need to be explored further. PGC-1β upregulates apoptosis and/or autophagy-related TFs, such as *Atf3* and *Stat3*, subsequently triggering myopathy (Sopariwala et al., 2017). MLN4924 is a specific inhibitor of Nedd8-activating enzyme that promotes the expression of ATF3 to induce autophagy in esophageal cancer (Liang et al., 2020). These findings indicate that high *Aft3* expression promotes autophagy. *Nr4a1* interacts with adenine nucleotide transposase 1 at the inner mitochondrial membrane to induce autophagy. In addition, the interaction of *Nr4a1* with cytoplasmic p53 may contribute to the induction of autophagy (Pawlak et al., 2015). Nr4a1 promotes high fat-related endothelial dysfunction by promoting the CaMKII–Parkin–mitochondrial autophagy pathway (Li et al., 2018). Here we found that the expression of *Nr4a1* in denervated skeletal muscle is low, which suggests that the TF may be involved in muscle atrophy *via* other pathways during denervated muscle atrophy. *JunB* inhibits starvation-induced autophagy; conversely, inhibition of *JunB* expression induces autophagy (Yogev et al., 2010). The JunB–FBXO21–ERK axis regulates cell apoptosis and cartilage matrix metabolism in osteoarthritis by inhibiting autophagy (Lin et al., 2021). We here found that *Junb* is highly expressed in denervated skeletal muscle and plays complex roles in muscle atrophy. Overall, *Ppargc1a, Runx1, Atf3, Nr4a1,* and *JunB* are all related to autophagy, but their roles in muscle atrophy are complex and require further study.

### Transcription Factors Involved in Oxidative Stress

The identified TFs involved in oxidative stress were *Stat3, Stat5a,* and *Runx1*. Ventilator-induced diaphragm dysfunction is associated with mitochondrial oxidative stress and impaired calcium homeostasis, which is a downstream pathway of STAT3 (Dridi et al., 2020). Fibronectin type III domain-containing protein 5 attenuates cardiac hypertrophy induced by obesity by inactivating JAK2/STAT3-related cardiac inflammation and oxidative stress (Geng et al., 2019). *Stat3* deletion sensitizes cell to oxidative stress (Barry et al., 2009). Raloxifene attenuates cardiac remodeling and inflammation induced by pressure overload, and oxidative stress in heart failure by inhibiting IL-6/STAT3 (Huo et al., 2021). Telomere shortening and oxidative stress in aged macrophages lead to the impairment of STAT5a phosphorylation (Sebastian et al., 2009). Attenuation of *Stat5a* is sufficient to enhance basal oxidative stress in normal CD34^+^ and chronic myeloid leukemia cells (Casetti et al., 2013). Further, down-regulation of RUNX1 induces extensive oxidative stress in mammary epithelial cell, which prevents cell proliferation and restores normal acinar morphology (Wang et al., 2011). Knockout of *Runx1* protects H9c2 cells against oxidative stress and apoptosis (Li and Jia, 2022). These findings indicate complex roles of these TFs in oxidative stress. The current study confirmed that oxidative stress plays an important role in denervated muscle atrophy. However, further research is needed to determine how Stat3, Stat5a, and Runx1 regulate oxidative stress and participate in muscle atrophy during denervated muscle atrophy.

### Transcription Factors Involved in Regeneration

The identified TFs involved in regeneration mainly included *Stat3* and *Klf5*. In mouse, *Stat3* promotes myogenic lineage progression of muscle stem cell by stimulating mitochondrial respiration (Sala et al., 2019). IL-6/STAT3 signaling regulates satellite cell behavior and promotes myogenic lineage progression by impacting *Myod1* regulation (Tierney et al., 2014). S1P promotes skeletal muscle regeneration by activating quiescent muscle satellite cell *via* the S1P2/STAT3-dependent pathway. Conversely, Stat3 negatively regulates the proliferation of muscle satellite cell and injury-induced muscle regeneration (Zhu et al., 2016). KLF5 is a zinc finger TF that is crucial for skeletal muscle development and regeneration (Zhang D.-H. et al., 2020). During muscle regeneration, *Klf5* is expressed in the nucleus of differentiated myoblast and newly formed myogenin-expressing myofiber. *Klf5* knockout inhibits the induction of muscle differentiation-related genes, including myogenin (Hayashi et al., 2016). Further, *Klf5* deletion seriously impairs muscle regeneration, and myotube formation is inhibited in *Klf5*-deficient C2C12 myoblasts and satellite cells (Hayashi et al., 2016). These observations indicate that Klf5 plays a beneficial role in muscle regeneration. Herein, *Klf5* expression is upregulated in denervated muscle atrophy, which may contribute to muscle regeneration. Further studies are needed to elucidate how *Klf5* regulates denervation-induced muscle atrophy.

## Conclusion

In the current study, using transcriptomics and bioinformatics analyses, we showed 24 h after denervation may be a critical turning point in muscle atrophy, which is consistent with our previous finding. Target genes of differentially expressed other TFs are mainly involved in metabolism, while those of differentially expressed known muscle-related TFs are mainly involved in the immune response. Correlation analysis between TFs and target genes in common, we identified 18 differentially expressed TFs closely related to the pathophysiology of skeletal muscle and constructed a functional network map based on these data. Our findings indicate that these TFs are mainly involved in inflammation, development, aging, proteolysis, differentiation, regeneration, autophagy, oxidative stress, atrophy, and ubiquitination. It should be mentioned that there are some limitations in this study. We mainly analyzed the gene expression patterns of known TFs related to muscle pathophysiology. Therefore, we might ignore some TFs that have not been deeply investigated but play a key role in denervated muscle atrophy. We will analyze the unknown TFs to shed light on new mechanisms in future’s work. Overall, these findings further revealed the molecular regulatory mechanisms in denervated muscle atrophy, and suggest potential targets for the prevention and treatment of muscle atrophy.

## Data Availability

The datasets presented in this study can be found in online repositories. The name of the repository and accession number can be found below: https://www.ncbi.nlm.nih.gov; GSE201025.
