# Supplementary figures and images for: Changes of Gene Expression Patterns of Muscle Pathophysiology-Related Transcription Factors During Denervated Muscle Atrophy
Source: Front Physiol. 2022 Jun 24;13:923190. doi: 10.3389/fphys.2022.923190 (PMC9263185; doi:10.3389/fphys.2022.923190)

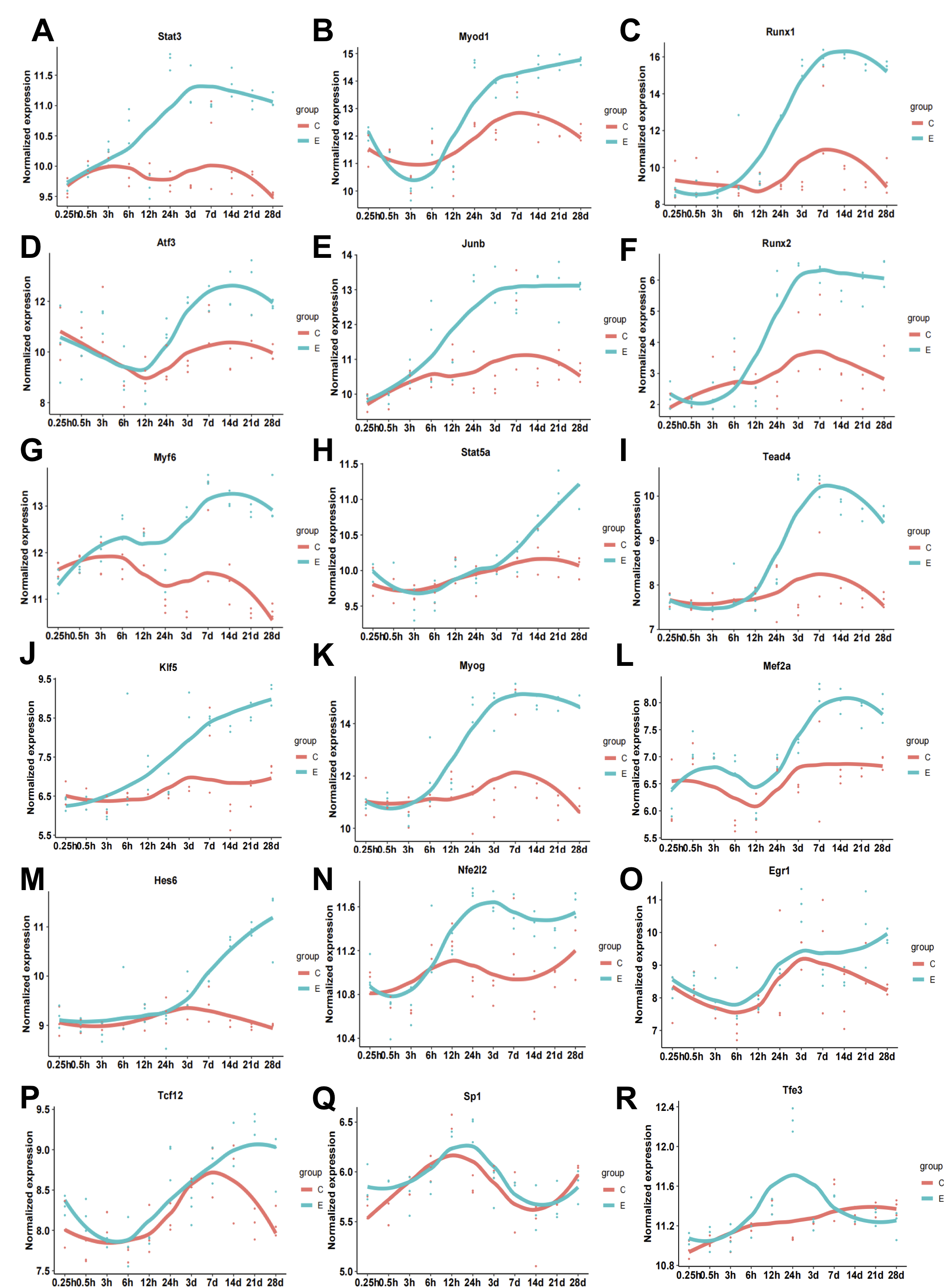

Supplement: Supplementary file 1 [file Image1.TIFF]

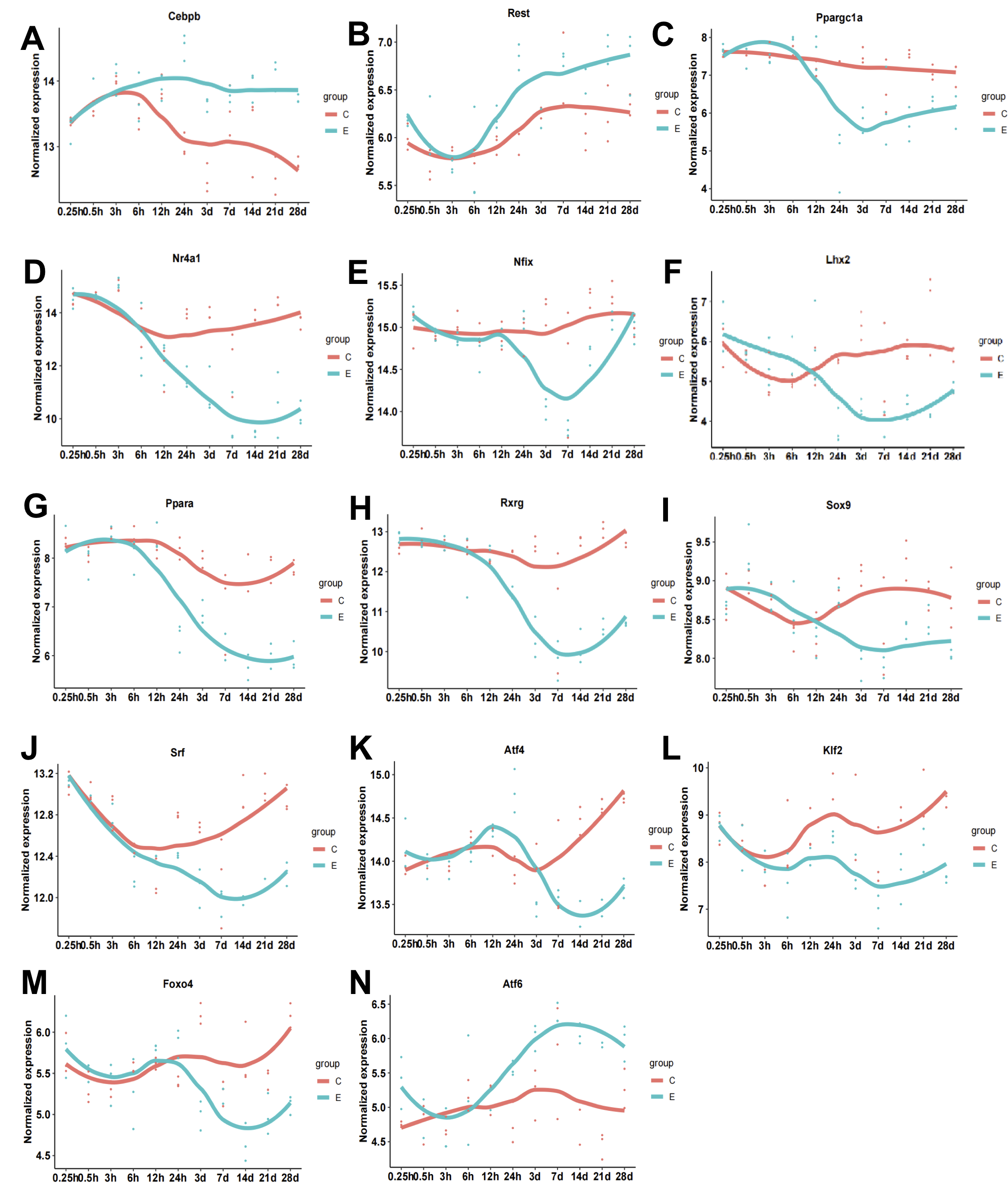

Supplement: Supplementary file 4 [file Image2.TIFF]
