# Supplementary material for: Changes of Gene Expression Patterns of Muscle Pathophysiology-Related Transcription Factors During Denervated Muscle Atrophy
Source: Front Physiol. 2022 Jun 24;13:923190. doi: 10.3389/fphys.2022.923190 (PMC9263185; doi:10.3389/fphys.2022.923190)
Supplement: Supplementary file 2 [file DataSheet1.PDF]

Supplementary Material

1 Supplementary Figures and Tables

1.1 Supplementary Figures

Supplementary Figure 1. Time-dependent expression of differentially expressed genes of 18 transcription factors.

Supplementary Figure 2. Time-dependent expression of differentially expressed genes of 14 transcription factors.

1.2 Supplementary Table

Supplementary Table 1. List of transcription factors related to pathophysiological processes of skeletal muscle.
